# Using gene panels in the diagnosis of neuromuscular disorders: A mini-review

**DOI:** 10.3389/fneur.2022.997551

**Published:** 2022-10-12

**Authors:** Kay W. P. Ng, Hui-Lin Chin, Amanda X. Y. Chin, Denise Li-Meng Goh

**Affiliations:** ^1^Division of Neurology, Department of Medicine, National University Hospital, Singapore, Singapore; ^2^Division of Genetics and Metabolism, Department of Paediatrics, Khoo Teck Puat - National University Children's Medical Institute, National University Hospital, Singapore, Singapore; ^3^Department of Paediatrics, Yong Loo Lin School of Medicine, National University of Singapore, Singapore, Singapore

**Keywords:** neuromuscular disorders, next-generation sequencing, gene panels, genetic testing, diagnosis, precision medicine

## Abstract

The diagnosis of inherited neuromuscular disorders is challenging due to their genetic and phenotypic variability. Traditionally, neurophysiology and histopathology were primarily used in the initial diagnostic approach to these conditions. Sanger sequencing for molecular diagnosis was less frequently utilized as its application was a time-consuming and cost-intensive process. The advent and accessibility of next-generation sequencing (NGS) has revolutionized the evaluation process of genetically heterogenous neuromuscular disorders. Current NGS diagnostic testing approaches include gene panels, whole exome sequencing (WES), and whole genome sequencing (WGS). Gene panels are often the most widely used, being more accessible due to availability and affordability. In this mini-review, we describe the benefits and risks of clinical genetic testing. We also discuss the utility, benefits, challenges, and limitations of using gene panels in the evaluation of neuromuscular disorders.

## Introduction

Neuromuscular disorders consist of a genetically and phenotypically heterogenous group of diseases, disrupting any component of the neuroaxis of the peripheral nervous system. These disrupted components can be skeletal muscle, neuromuscular junction, or nerves. The causes can be genetic (single gene disorder, polygenic disorder), nongenetic (infective, autoimmune, autoinflammatory), or yet to be identified.

Prior to the availability of genetic testing, neurophysiology and histopathology were the go-to primary investigations. The advent of single gene testing in the 1990s (Sanger sequencing) allowed for better diagnostic capabilities, but it was fraught with issues. It was costly, not widely available, laborious, and had a long turnaround time. As such, clinicians tended to test one gene at a time. They often had to fret over the test sequence of the candidate genes; this was especially challenging for neuromuscular disorders because of their genotypic and phenotypic heterogeneity.

This problem was alleviated with the discovery of next-generation sequencing (NGS). NGS is a high-throughput method that allows for the evaluation of many genes in a single reaction, resulting in a faster turnaround time, better diagnostic yield, and lower cost than Sanger sequencing (1). NGS and other advances have led to the discovery of many disease genes for neuromuscular disorders (2, 3), hence allowing for advancement in achieving a diagnosis and understanding the pathogenesis, paving the way for personalized medicine and the development of targeted gene therapies for previously incurable diseases (4).

There are currently >600 single gene disorders that are known to cause neuromuscular diseases (3). Historical surveys suggested an approximate prevalence of 33/100,000 for inherited neuromuscular disorders (5, 6), but with the increasing rate of genetic diagnosis, the prevalence rates have increased to around 160/100,000 population, being almost on par with Parkinson's Disease (100–300/100,000 population) (7).

Nowadays, the clinician has the opportunity to utilize various forms of clinical genetic tests e.g., gene panels, whole exome sequencing (WES), and whole genome sequencing (WGS) (8). Gene panels are often the most widely used, being both clinically available, widely available, and more affordable. In this mini-review, we describe the benefits and risks of clinical genetic testing. We also discuss the utility, benefits, challenges, and limitations of using medical-grade gene panels in the evaluation of neuromuscular disorders.

## Benefits of genetic testing

Genetic testing can be done using a medical-grade test, a research-grade test, a direct-to-consumer test (DTC), etc., In this review, the term “genetic testing” is used to refer to using medical grade tests. Medical-grade genetic tests are tests done in certified laboratories, hence ensuring minimal standards in quality control and quality assurance.

There are many benefits to genetic testing. Genetic testing in neuromuscular disorders allows for more patients to achieve a definitive molecular diagnosis. A diagnosis gives the patient closure, as well as access to better information on the condition, prognosis, progression, treatment, and risk of transmission/recurrence.

The use of genetic testing as a first-line test shortens the diagnostic odyssey and minimizes diagnostic delays. Patients can also avoid unnecessary and/or invasive investigations, such as muscle or nerve biopsies (9–12). When Haskell et al. reviewed undiagnosed neuromuscular patients who had undergone investigations prior to targeted exome sequencing, they found that nearly 40% of the procedures, including muscle biopsy, were not helpful with pinpointing a molecular diagnosis in the patients (13). These benefits of early genetic testing have positive impacts on the patient's psychological well-being (14–16). They also result in cost-savings (17).

A molecular diagnosis allows for more appropriate surveillance. For example, patients who are diagnosed with a Limb-Girdle Muscular Dystrophy (LGMD) subtype known to be associated with cardiac complications, they will benefit from pre-symptomatic cardiac screening and treatment (18). Patients diagnosed with LGMD subtypes that are not associated with cardiac complications can do without additional cardiac appointments and cardiac tests.

A molecular diagnosis allows for more appropriate treatment. For example, patients with certain forms of spinal muscular atrophy (SMA) can access SMA therapeutics, where earlier diagnosis and earlier treatment leads to a better outcome (19–21). In congenital myasthenic syndromes, knowing the gene and the variant can help the physician select the more effective pharmacotherapy and avoid medications that are harmful or ineffective (22). In many forms of muscular dystrophies, patients with a molecular diagnosis can avoid unnecessary, and potentially harmful, empiric treatments such as immunosuppression (18).

A molecular diagnosis allows for better access to clinical trials and emerging treatment modalities. Currently, the eligibility criteria for many clinical trials and FDA-approved personalized therapies require the information of the individual's molecular diagnosis. With accelerating targeted drug development for genetic causes of amyotrophic lateral sclerosis (ALS), early genetic testing is now recommended for all ALS patients to ensure access to therapeutic intervention (23, 24).

Knowledge of the causative variant and its mode of inheritance enables family planning, predictive testing of at-risk family members, and carrier testing of family members. Another potential benefit of earlier diagnosis is better natural history studies, especially for some of the disorders that do not have the classic clinical phenotype. For more information, the reader can read the consensus statement by the American Association of Neuromuscular and Electrodiagnostic Medicine (AANEM) on the utility of genetic testing in neuromuscular disorders (25).

## Risks of genetic testing

There are risks and limitations to genetic testing. To the patient, these risks include cost, anxiety, stress, depression, guilt, as well as adverse effects on insurance, employment, and other family member's health and insurance. For the clinician, inappropriate testing, inappropriate test interpretation, inappropriate follow-up action by the clinician can result in harm to patient, as well as potential risks for patient complaints, medicolegal actions, and increased cost to the health care system. Hence, first, do no harm. If in doubt, the clinician should consider referring the patient to the appropriately trained personnel (eg. neurogeneticist, clinical geneticist) to help them with genetic testing.

## Determining if a genetic test is indicated

The clinician is faced with the challenge of deciding whether to pursue a genetic test vs. doing other laboratory tests to evaluate for nongenetic causes. In most situations, the clinician is considering whether to use a genetic test for diagnostic purposes i.e, the patient has symptoms, and the clinician is trying to find the cause. Genetic testing is also sometimes used for predictive testing or carrier testing purposes.

Some common neuromuscular phenotypes that may trigger genetic testing by the physician include:

- Myopathic phenotypes (eg. congenital dystrophies, muscular dystrophies, congenital myopathies, metabolic myopathies, mitochondrial myopathies, myotonic dystrophies, periodic paralyzes, distal myopathy, limb-girdle muscular dystrophy)
- Isolated persistent/recurrent high creatine kinase (CK)
- Abnormal neuromuscular junction transmission phenotypes (eg. congenital myasthenic syndromes)
- Neuropathic or motor neuronopathy phenotypes (eg. Charcot–Marie–Tooth Disease, hereditary neuropathy of pressure palsy, spinal muscular atrophies, amyotrophic lateral sclerosis)
- Upper motor neuron phenotypes (eg. hereditary spastic paraplegias)
- Associated with Syndromic/Multisystemic involvement (i.e., involving more than 1 organ system)

° eg. dysmorphism
° eg. neurological system: encephalopathy, global developmental delay, intellectual disability, epilepsy
° eg. growth: growth failure, overgrowth syndrome
° eg. renal/urological tract: malformations, chronic kidney disease
° eg. digestive/liver involvement

Of these, certain phenotypic characteristics are more likely to be associated with a single gene disorder. These include:

- Positive family history or consanguinity (11, 26, 27).
- Younger age of onset (11, 26, 28–30).
- Abnormal investigations associated with a myopathic phenotype [eg. abnormal Magnetic Resonance Imaging (MRI) findings of the muscle, abnormal electromyogram findings, and abnormal muscle histopathology (30)].

The clinician needs to bear in mind that a phenotypic diagnosis can sometimes be wrong. For example, Anti-HMGCR myopathy can mimic LGMD (31), while Charcot–Marie–Tooth disease can be misdiagnosed as chronic inflammatory demyelinating neuropathy (17).

The clinician should also know when diagnostic genetic testing is not indicated. For example, gene testing should not be used for the diagnosis of ALS (27), which should instead be based on clinical criteria (32). This is due to the complex and not fully characterized contribution of genetics to the development of ALS (27). One of the reasons genetic testing can be done in ALS is for the purpose of identifying the causative variant for predictive testing in other family members. Hence, genetic testing is complex because the clinical indications (diagnostic, predictive, carrier testing) can also influence whether genetic testing is warranted or not.

If the clinician wants to choose a genetic test, it is usually a choice between specific variant testing, common variant(s) testing, single gene testing, gene panel, WES, or WGS. This is one of the hardest decisions for a clinician to make as it requires domain knowledge about multiple areas including test methodologies and the genetics of the condition. A gene panel, though cheaper and faster, may not always be the best first-line test. Just because genetic testing is more widely available does not mean every practitioner should send genetic testing. The first rule of medicine applies—only order when you have the clinical expertise, and when you know how to interpret the results. Otherwise, discussion and collaboration with the relevant specialists is the wisest choice.

## What is a gene panel

The human being has a nuclear genome and a mitochondrial genome. The nuclear genome refers to the double-stranded helical DNA found inside the nucleus of the cell. It is predicted to contain about 20,000–30,000 genes packaged into 46 chromosomes. The mitochondrial genome (mtDNA) refers to the circular DNA found inside the mitochondria of a cell (not in the nucleus). It has about 37 genes (33).

The term whole genome is used usually to refer to both the nuclear genome and the mitochondrial genome. However, when used in the context of “whole genome sequencing”, some labs use it to mean only the nuclear genome and not the mitochondrial genome, whereas other labs will use that term to encompass both genomes. This arises because different test methodologies are usually needed to study these two genomes.

The term whole exome is used to refer to all the exons that, when transcribed, remain within the mature RNA. The exome consists of 1–2% of the genome. Whole exome sequencing usually only involves the nuclear genome. It is predicted to contain ~85% of the variants detectable by whole genome sequencing (34).

A gene panel is a laboratory test that contains >1 gene, and the genes included in the panel were selected by the lab based on what the lab deemed relevant to a particular phenotype. Eg. myopathy gene panel, muscular dystrophy gene panel, and inherited neuropathy gene panel. A gene panel is hence usually testing for a subset of the exome and a subset of the genome.

For a similar phenotype, the contents of a gene panel can vary between different laboratories. For many years, there was limited international consensus or guidelines on what genes should be included in the phenotype-related gene lists (35). This resulted in wide differences between labs. In recent years, the ClinGen consortium has put forward recommendations to try to enhance utility (https://clinicalgenome.org/). This has reduced the variability, but differences remain. Eg. In lab A, their muscular dystrophy panel consists of 56 genes. In lab B, their muscular dystrophy panel consists of 60 genes. Some labs do not allow the ordering health care worker to customize their panels while others do, such as by adding in genes or removing genes.

Gene panels vary in size depending on the phenotype tested (ranging from a small number to a large number of genes). For example, the spinal muscular atrophy panel consists of 2 genes, whereas a comprehensive neuromuscular panel may consist of up to 230 genes or more. Some labs price their gene panels at one price regardless of the size, while others price their gene panels according to their sizes.

There are also other terminologies used interchangeably to describe gene panels. Some will use “focused” gene panel, “targeted” gene panel, or “multi-gene” panel; these terms in essence all relate to a test that consists of a set of genes that are simultaneously tested. Some will use focused, narrow, or small to describe gene panels that contain a small number of genes. Some will use large, broad, or comprehensive to describe gene panels that contain many genes. There is no standardized definition of what constitutes these terms; they are purely arbitrary.

The predominant technology used in gene panels is next-generation sequencing (NGS). NGS is a massively parallel sequencing technology that allows for high throughput, scalability, and speedy testing. Some gene panels solely use NGS, whereas other gene panels use NGS plus other methods to fill in the gaps inherent to NGS methodology. Some gene panels involve NGS and analysis of the included genes only, whereas others are exome-based tests where NGS of the exome is completed but a subset gene analysis is done and reported as a gene panel.

## Benefits of using gene panels

There are many benefits of using gene panels.

NGS-based gene panels are less costly than traditional Sanger sequencing. They are also less costly than WES and WGS.

Gene panels can simultaneously assess multiple genes associated with a phenotype (36, 37). This allows for faster diagnosis compared to sequential Sanger sequencing of genes (8). Gene panels can also study genes that could not previously be effectively tested e.g., very large genes relevant to muscle diseases, such as *TTN, NEB, RYR1*, and *DMD* (38).

Gene panels are generally designed to ensure good coverage of the genes i.e., all regions of interest are well tested. Gene panels may have additional measures put in place to help pick up changes missed by the NGS platform eg. they may have been supplemented by other methods to enable the detection of large deletions or duplications, repeat expansion disorders, repeat contraction disorders, or mosaicism (39–43).

The use of gene panels allows for diagnosis when there is a limited skill in genotype–phenotype correlation, for example, access to gene panel testing can allow for diagnosis when there is a lack of specialist clinicians who are able to discern candidate genes and formulate a gene test set based on proband phenotype (44, 45).

The use of large gene panels may also lead to unexpected findings that broaden the phenotypic spectrum associated with specific genes, furthering knowledge into the heterogeneity of neuromuscular diseases (11, 44).

In comparison to WES or WGS, gene panel testing has a lower risk of obtaining uncertain, secondary, or incidental findings that may not be related to the presentation triggering the genetic testing (46).

All these benefits make gene panels a reasonable first-tier approach in the genetic screening of patients with neuromuscular disorders (41, 47–51), providing more individuals with neuromuscular disorders with the opportunity to attain a molecular diagnosis.

## Disadvantages of a gene panel

Gene panels cover some genes but not all genes in the human being. So, if the gene is not on the panel that gene is not evaluated. This has implications on how one interprets a negative result (i.e. is this a true negative or a false negative result). This is discussed in the later sections of this review.

Pure NGS-based gene panels are not able to evaluate certain parts of the human genome eg. repeats, highly homologous regions, and regions of high and low GC content (37). Common neuromuscular repeat expansion disorders not detectable *via* NGS include myotonic dystrophy 1 and 2, facioscapulohumeral muscular dystrophy 1 (FSHD1), oculopharyngeal muscular dystrophy (OPMD), oculopharyngodistal myopathies (52), and *C9orf72*-related amyotrophic lateral sclerosis. When ordering a gene panel, it is thus essential to check if the gene panel included the appropriate methodology to cover these special conditions. For example, in some laboratories, if their gene panel for the ALS phenotype is only NGS-based, *C9orf72* for hexanucleotide repeat expansion will not be performed. If you chose that gene panel, you would need to separately order the C9orf72 repeat expansion assay. On the other hand, other laboratories may have an ALS gene panel that by default is already a combination of an NGS-based test and a *C9orf72* hexanucleotide repeat expansion assay.

Pure NGS-based gene panels have variable sensitivity for detecting copy number variations (CNVs). CNVs are structural changes of the genome, in which a certain genomic sequence is present at a different copy number compared with the reference genome. CNVs can range from being as small as 50 base pairs to being very large (53). CNV analysis is important when testing for conditions such as SMA, *PMP22*-related Charcot Marie Tooth, and female dystrophinopathy carriers (54, 55). Other techniques such as multiplex ligation-dependent probe amplification (MLPA), quantitative polymerase chain reaction, and cytogenetic genomic hybridization (CGH) arrays are needed to evaluate CNV. Some gene panels automatically include these other techniques, but some do not.

Deep intronic variants and large structural variants such as big inversions and translocations may be missed by NGS-based gene panels. Deep intronic variants or structural variants account for about 2% of the Duchenne Muscular Dystrophy (DMD) patient population (56). Muscle RNA sequencing analysis for transcription studies may be required to detect the molecular mechanism in such patients (57).

NGS-based gene panels cannot detect epigenetic phenomena. Epigenetic phenomena are non-DNA changes that affect gene function such as methylation that changes the protein coating of a gene. Transcription analysis, which is the study of the way genes are transcribed to synthesize functional RNA species or protein products, may be required to detect such changes. For example, the discovery of epigenetic changes helped us understand the role of recessive *RYR1* variants in congenital myopathy (58).

When a mitochondrial disorder is suspected, concurrent testing of both mtDNA and nuclear mitochondrial genes is usually recommended to prevent diagnostic delays. Many gene panels only cover genes in the nuclear genome; they do not evaluate the mitochondrial genome; and vice versa. It is also important to note that some laboratories offering whole exome or whole genome tests may not automatically cover the mitochondrial genome either. Testing should ideally be done using the affected tissues (eg. muscle in mitochondrial myopathies instead of blood) for a higher yield due to heteroplasmy (59–61), though in practice, it is logistically easier to use blood samples.

Gene panels can be outdated if they are not routinely updated. It may be technically easier to update the curated gene list in a WES/WGS-based test than periodically designing a new gene panel, which may also aid in future data reassessment. Gene panels usually have a fixed turnaround time. Most labs do not have a stat or expedited turnaround time for panels. On the other hand, WES and WGS frequently have stat and expedited turnaround time (usually at additional cost).

## When to choose a gene panel

There is no perfect genetic test. When choosing a genetic test, the clinician must first consider whether they have the competence to make that decision. If they are not competent in how to choose a genetic test or how to interpret the results, the clinician and their patient would benefit from a referral to the appropriate specialist (e.g., a neurogeneticist, a clinical geneticist).

If the family's variant is already known, single targeted variant testing may be the most appropriate test. In diseases where the phenotype is easy to recognize and is known to be due mostly to one variant, single targeted variant testing may be more efficient and cost-effective than multigene testing. For example, most patients with demyelinating Charcot–Marie–Tooth disease have a *PMP22* copy number variant. It may be more cost-effective to start with a *PMP22* duplication test rather than a multigene panel (46). This *PMP22* duplication test has the highest yield in patients with a definite neuropathy subtype as defined by electrodiagnostic data and who are also evaluated by specialized neuropathy clinics (44).

In situations where there is a known high prevalence of founder variants among well-studied ethnic groups (37, 62), testing for a set of common variants may be the most appropriate. If the disease has minimal locus heterogeneity, single gene testing may be more suited than a gene panel (63).

For most patients, however, the above situations do not apply. Even with a well-defined phenotype (eg. neuropathic, myopathic, hereditary spastic paraplegias), the patient is usually the first affected in the family, and the gene list associated with that phenotype is usually long. In such individuals, the diagnostic yield of gene panels is good, therefore, making gene panels suitable as the first-tier approach (13, 28). This gene panel-first strategy has recently been shown to be cost-effective (10, 64). If the gene panels are not informative, the second tier test is WES/WGS (65). This approach, however, may evolve with time as cost and accuracy of WES improves.

## When not to use a gene panel

In clinical practice, challenges in phenotyping may lead to difficulty in choosing the appropriate gene panel. Phenotypic characterization can be difficult due to variable disease penetrance and expressivity (66), or due to early, evolving, or atypical presentations (28). It is also often difficult to characterize the mode of inheritance in small families or when there is no family history (67).

When a patient has dual pathology eg. neuromyopathies causing distal weakness, the choice of gene panels for testing can be complicated (30, 68, 69). Diseases with poorer genotype–phenotype correlation may have a lower diagnostic yield with gene panels. Phenotypes that are multisystemic or atypical have a lower diagnostic yield with gene panels (30, 44). WES may, therefore, be more helpful in such patients (13, 70, 71).

WES that includes the mitochondrial genome has been described to potentially have a diagnostic edge over targeted gene sequencing. Though traditionally WES had less depth of coverage than gene panels, WES can now detect >98% of pathogenic mutations identified on targeted NGS gene panels (72). Furthermore, many laboratories now sequence WES at comparable depths to gene panels. WES also has the added benefits of future data reassessment (73), trio-sequencing for better yield and variant clarification (74), availability of upgrading to a faster turnaround time, and enables novel gene discovery. With declining cost, WES may be an increasingly attractive alternative to gene panels since gene panels can become outdated quickly.

Other than WES, WGS is also an alternative to gene panels. WGS can identify deep intronic variants, large structural variants, and better assess some coding regions than WES (75) offering an increased diagnostic yield over WES (76). For example, WGS when used in previously genetically undiagnosed dystrophinopathies has identified deep intronic or complex structural variants (77, 78). WGS is currently not as widely available as WES. High costs and complexity of data analysis and variant interpretation also currently limit its usage.

Not all diseases can be solved by genetic testing. Polygenic diseases such as chronic axonal polyneuropathies have reduced diagnostic yield on gene panel testing (11). They may also have a low yield with WES/WGS.

## How to choose a good quality gene panel

Here are some tips on how to choose a good-quality gene panel.

**i Use an accredited lab and one that has expertise in the area that you are interested in**. Accredited labs are likely to have well-designed diagnostic gene panels that meet the technical standards of the American College of Medical Genetics and Genomics (ACMG) (37). Their gene panels are more likely to be optimized for clinical sensitivity by including all genes associated with a disorder as well as genes associated with disorders with overlapping phenotypes which constitute the differential diagnoses.
**ii Look at the content of the panel and check if it contains the genes that you are most interested in. Genes may also need to be included to cover the possibility of variable expression and atypical phenotypes** (63).
For example, when testing for causes of rhabdomyolysis and exercise intolerance, the gene panel may need to include genes associated with metabolic myopathies (glycogen-storage disorders, fatty acid metabolic disorders, and mitochondrial respiratory chain disorders) (79), as well as genes not typically considered to cause a metabolic myopathy but which can result in similar clinical features, such as dystrophies (80), *RYR1* (81), or channelopathies (82).
A myopathy panel could also include genes relating to congenital myasthenic syndromes as they can mimic myopathy, such as *DOK7* (83). A distal myopathy panel may also want to include traditional LGMD genes *CAPN3* and *CAV3* as these can also result in a distal myopathy phenotype (84).
When testing a patient who has an LGMD phenotype, apart from the usual LGMD genes, the panel should also include genes that cause metabolic myopathies that may present with a fixed LGMD phenotype such as acid alpha-glucosidase deficiency, CPT2 deficiency, and McArdle disease (83, 85).
**iii With the advent of targeted therapies available for specific genetic diseases, it is also important to ensure that the selected gene panel contains treatable disease genes with phenotypic similarity**. Eg. in a patient with muscle weakness, genes for Pompe Disease, Congenital Myasthenic Syndrome (52), and Spinal Muscular Atrophy (86) should be included to avoid missing a potentially treatable disease.
**iv Check the methodology used and see if it will cover the molecular mechanism that you are interested in**. Eg., Are repeat analysis or copy number variant analysis important considerations? Some gene panels include auxiliary assays for genes or regions that cannot be fully assessed by the NGS technology of a gene panel (11, 40–43, 63, 87), while others do not. You can often find this information in the section where the lab states its methods and its diagnostic limitations of the panel (37).
**v Choose a gene panel that includes an alternative method for confirmation/validation of the variant identified**. False-positive results in NGS techniques can occur, though this is rare especially if a lab uses an alternative method to confirm the presence of the variant (88). You can often find this information in the section where the lab states its methods.
**vi For tests that are similar, look at their price point, check whether they are covered by the patient**'**s insurance provider, and look also at the turnaround time of the tests**. These factors may influence your choice of the laboratory and test.

With gene panel testing, there may also be ongoing and evolving technical considerations that require close collaboration and regular discussion with the geneticists.

## Diagnostic yields of gene panels

Overall, diagnostic yields depend on the characteristics of the study cohort (eg. size of cohort, levels of consanguinity, age of onset, extent of prior investigations and specialist work-up, and heterogeneity of phenotypes), as well as the strategies employed in the usage of the gene panel (eg. when to use, testing methodology, size, and composition of gene panel).

In the studies using comprehensive gene panels for undifferentiated categories of neuromuscular disorders, the yield has ranged from 12.9 to 48.8% (13, 28, 41, 43).

For muscle disorders, diagnostic yield can range from 13 to 79% depending on the selected study cohort and gene panel testing techniques (10, 12, 13, 30, 84, 87, 89). For well-defined myopathies, a narrower gene panel may have comparable diagnostic yields as a broader gene panel (13). For more complex or nonspecific muscle phenotypes, the diagnostic yield is lower (85). However, a recent study using a gene panel showed a positive diagnostic yield of 50% in patients with pauci- or asymptomatic hyperCKemia (90).

For neuropathies, gene panels have been reported to have a positive diagnostic yield of 6%−46% in inherited neuropathies (11, 26, 44, 91–95). For well-defined neuropathic phenotypes, a narrower gene panel may have comparable diagnostic yields as a broader gene panel (13). Hereditary spastic paraplegias have a reported diagnostic yield of more than 20% for NGS approaches (96–102). Demyelinating neuropathies have better yield compared to axonal or mixed neuropathies, even after *PMP22* deletion or duplications have been excluded (11, 26). However, for unexplained axonal polyneuropathy, even in those with a family history, the yield of gene panels was much lower; 7% in patients with family history and 2% in those without (103).

For ALS, Shepheard et al. found that prospective genetic screening of 100 patients diagnosed with ALS using an ALS gene panel led to clinically actionable results in ~21% of patients, and a further ~21% of patients were found to have a variant of unknown significance (VUS) (104).

Patients with more complex phenotypes or phenotypic overlap, benefit more from broader or more comprehensive gene panels than with small gene panels (13, 28, 29, 43, 47, 64, 105). Krenn et al. reported a diagnostic yield of 34.7% with comprehensive gene panels (up to 344 genes) as compared to 22.2% with small gene panels (417 genes) (105). Integrating SMA analyses into a multigene neuromuscular disorders panel improved diagnostic yield (29, 86, 106).

## Diagnostic yield of gene panels compared to sequential single-gene testing or WES

In general, targeted gene panel analysis has a higher diagnostic yield when compared to sequential single gene testing. There is a 3-fold increase in diagnostic yield for neuromuscular diseases (41). There is a 6- to 10-fold increase in diagnostic yield for rarer genetic subtypes of Charcot–Marie–Tooth disease (26). The use of gene panels for familial ALS patients resulted in the detection of potentially pathogenic variants in 45.5% of patients compared to 23.8% when Sanger sequencing was used (107).

Comprehensive neuromuscular gene panels have been reported to have comparable or slightly lower diagnostic yield than WES (10, 11, 41, 46, 72, 105, 108–111). Narrower gene panels, however, may not perform as well as WES and may incur more accumulative cost in the long run for the patient (28, 47).

## Pretest genetic counseling and written consent is usually required

For many germline tests, written consent is required. This written consent is usually taken after appropriate pretest genetic counseling (see section 15).

## Challenges with interpreting the results of a panel

Most lab reports come with interpretations of the results. Some are well written and complete; others are not so well written and may leave out important information. Decoding/interpretation of genetic test results can be challenging. Even though it is written in English, the jargon and nomenclature used require understanding and fluency. For those who want to learn more, ClinGen has some useful resources https://www.clinicalgenome.org/tools/educational-resources/materials/introduction-to-variants-and-nomenclature/.

Prior to 2015, there were no widely accepted guidelines on how to classify variants. As such, there was discordance in variant classifications between labs and inaccurate variant calling. In 2015, the American College of Medical Genetics and Genomics (ACMG) and the Association for Molecular Pathology (AMP) introduced guidelines to help standardize this process. These guidelines, however, were not formulated for calling copy number variants or for somatic/cancer variant calling. Other guidelines were subsequently formulated to cover these areas.

The ACMG-AMP's guidelines for variant classification is the one that neurologists will most likely encounter. This guideline categorizes a germline genetic variant as a pathogenic, likely pathogenic, variant of uncertain significance, likely benign, or benign (112). A simplified definition list is shown below:

- **Pathogenic**: the variant is known to be disease-causing
- **Likely pathogenic**: there is greater than 90% certainty that a variant is disease-causing
- **Variant of uncertain significance (VUS)**: variant's effect on health or disease is uncertain
- **Likely benign**: there is greater than 90% certainty that the variant is not disease-causing
- **Benign**: the variant is not disease-causing

Most genetic testing reports will provide the ACMG-AMP criteria used to reach that variant's classification (eg. prevalence in disease, population frequency, bioinformatic prediction of the variant's effect). Concordance of interpretation of variants across different laboratories has been reported to be at only 71% (113). There is some subjectivity to the ACMG-AMP guidelines, and hence laboratory geneticists can apply the guidelines differently. Some variants may be misclassified (114).

Reclassification of the variant can occur. This tends to happen over time as more knowledge is gained. Variants can be upclassified or downclassified. For example, some variants initially classified as pathogenic or likely pathogenic have been downclassified in some cohorts of patients with LGMD (115) and dysferlinopathy (116). Some labs take on the responsibility to periodically review past patients and send updated reports to the ordering doctor when there is a reclassification. Others do not and leave the responsibility of reviewing the variant classification with the patient's doctor.

Discerning the applicability of genetic test results to the patient requires expertise in genomics not only on the part of the laboratory geneticist but also from the clinician (117). Mistakes that have occurred include:

- Not understanding the difference between heterozygote, compound heterozygote, homozygote, and hemizygote, resulting in wrong interpretation and wrong treatment of patients;
- Not understanding what it means when the lab says they do not know if two variants detected are in *cis* or *trans*, and wrongly assuming that the patient is a compound heterozygote;
- Not understanding the difference between a silent carrier and a presymptomatic individual;
- Misinterpreting the implications of a variant of uncertain significance;
- Assuming that a negative report is a true negative without consideration for the possibility of false negative results or residual risk;
- Assuming that a polymorphism is always benign;
- Assuming that a rare variant must cause disease;
- Attributing a variant to a disease because of outdated information found in old publications and not realizing that the variant has been reclassified as benign.

Do consider communicating with the testing laboratory for clarifications if there are doubts, questions, or difficulties in interpreting the reports. Consider a referral to the appropriate specialist (eg., a neurogeneticist, a clinical geneticist) if additional assistance is required in assessing the applicability of the genetic test result to the patient and if this was not already previously done.

### Challenges interpreting a positive report (pathogenic variant and a likely pathogenic variant)

Many labs will use the term “positive” report when a pathogenic or likely pathogenic variant has been identified. In addition to looking at the variant classification, a positive report must also be interpreted in the context of the zygosity of the variant, the phase of the variant, the inheritance pattern of the disorder, the molecular mechanism of the disease, and the patient's phenotype.

Zygosity can be

- **Heterozygous:** the gene comes in a pair; at that position in the DNA sequence, there is one normal variant, and the other variant is different from the reference sequence.
- **Homozygous:** the gene comes in a pair; at that position in the DNA sequence, the pair of variants are identical to each other but different from the reference sequence.
- **Hemizygous:** this gene is on the X chromosome; this term is used to describe a male patient who only has one copy of that gene, and at that position in the DNA sequence the variant is different from the reference sequence.

It is also important to consider the mechanism of the disease when interpreting results. In an autosomal dominant condition, a pathogenic or likely pathogenic variant is usually sufficient to cause disease. However, this is not always the case. For example, *SMCHD1* is included in some dystrophy panels. Pathogenic variants in this gene by itself are not sufficient to result in FSHD. In FSHD1, contraction of the repeat by itself is insufficient to result in FSHD; the repeat contraction needs to occur on a permissive allele.

In a recessive condition, if the patient is homozygous for a likely pathogenic or pathogenic variant, this is in keeping with the disease mechanism. However, one should never assume that the parents are carriers. Parental testing is still needed to prove that they are carriers (as the second variant can rarely arise from spontaneous mutation or due to uniparental isodisomy).

For recessive conditions, you may sometimes come across this phrase “there were two variants identified but we are not able to determine the phase of the variants”. Or, you might see this phrase “there were two variants identified but we are not able to determine if the variants are in cis or in trans”. “Phase” refers to the physical relationship between the variants. In current-day NGS, the DNA sequencing output comes in short reads (segments), which are then assembled into a long read out using bioinformatics. Thus, when we see in the same gene two variants that are physically far apart from each other, we are not able to determine whether the variants are on the same chromosome (in *cis*) or on different chromosomes (in *trans*). This makes a difference in the way we ascertain if the variants are sufficient to cause disease, especially in recessive conditions. The phase is usually ascertained by doing variant testing in the parents, or sometimes, long-read DNA sequencing of the patient is used. If the two variants are on two different chromosomes, and the gene involved is a recessive disorder, then it is in keeping with the mechanism of the disease. If the two variants are on the same chromosome, then we have not identified the second variant as a recessive disorder. Some of the possible reasons why the second variant could not be found include (1) there really is no second variant i.e., the patient is only a carrier for a recessive disorder; (2) the second variant is present but could not be identified by this test's methodology. There are ways to further sort this out. This is complex and best discussed with the lab, clinical neurogeneticist, or a clinical geneticist.

In a female who has one likely pathogenic or pathogenic variant for an X-linked recessive condition, X inactivation pattern may affect the expression of the disease in her. X-inactivation patterns are usually not studied by gene panels.

Lastly, the clinician must determine if the patient's phenotype fits the gene identified. If it does not, please have a conversation with the lab reporting the test and/or a clinical expert.

Even with a positive result, the result may not always allow a prediction of disease course, penetrance, or expressivity (118).

### Challenges interpreting a variant of uncertain significance

Variants may be classified as uncertain if there is limited evidence available to predict their likely impact on gene function and disease. Additional tests may be required to clarify their clinical significance. This can include familial segregation, reverse deep phenotyping (119), neurophysiology, histopathology with immunostaining, Western blot for possible affected proteins, and neuroimaging such as muscle MRI to look for characteristic patterns of muscle involvement (119, 120).

When considering all genetic conditions, over time, there is a ~75% chance that a VUS is downgraded, and a ~25% chance that a VUS is upgraded (121). In one study on neuromuscular patients, reanalysis of probands with inconclusive results on WES revealed new diagnoses in 15.5% (122). The speed at which knowledge is updated is unfortunately limited by the ability for international collaboration and publication of data (123). Hence, periodic reevaluation of the VUS is imperative as variants get reclassified over time due to improving knowledge.

Reevaluation of a VUS requires skills and knowledge in genomics, in the use of databases, in the use of bioinformatics tools, as well as in how to rationalize conflicting data. These are highly complex areas where the novice is likely to make mistakes. The question then is whose job is it to reevaluate the VUS? Some labs have taken on that responsibility. They will periodically reevaluate the patients with VUS and will issue updated reports when there is reclassification. The onus is then left to the ordering doctor to recontact and inform the patient. This leaves the question as to whether the clinician has a duty of care to recontact and update the patient. Other labs do not take on the responsibility of reevaluation. We are then left with the question as to whether the ordering physician or the current physician has the responsibility to reevaluate the VUS. What if the physician does not have that expertise? In this situation, it may be best to contact the lab and ask for a reevaluation of the VUS. To deal with all the above complexities, a multidisciplinary team consisting of the neurologist, geneticist, pathologist, radiologist, and genetic counselor may be required (124).

If a VUS remains a VUS, the doctor needs to know what they can or cannot do based on those results. This is important as it has implications on patient and their family.

### Challenges interpreting a negative test report

What does a negative test report mean? It could mean

- The patient truly does not have a genetic cause (true negative)
- The cause has not yet been identified (false negative)
- The causative gene was not included in the test (false negative)
- The causative variant could not be detected by the test methodology (false negative)
- The causative variant could not be detected in the tissue used (eg., blood for mitochondrial depletion studies)

Hence, when there is a negative result, the doctor needs to consider the possibility of a false negative result and the possibility of residual risk.

If a genetic cause is still suspected in the event of a negative gene panel, several approaches can be taken. Additional tests that evaluate regions not well covered by the gene panel can be undertaken, such as Sanger fill-in, CNV analysis, and repeat analysis. Other approaches include WES, WGS, or other more complex testing, which are beyond the scope of this review (57, 125, 126).

## Complexities of genetic counseling

The complexity of genetic testing necessitates that there is appropriate pretest genetic counseling to ensure that the patient makes a thorough and informed decision about proceeding or not proceeding with genetic testing. This requires sufficient time and training. The points that are usually covered include:

- The aim of genetic testing
- The potential benefits
- The potential risks
- What the test options are, what each test can do and what it does not do
- What the alternatives are to genetic tests
- The option of not testing and its implications
- The costs and turnaround time
- The potential results and their implications eg. Any potential change in current management, any potential future management
- The potential effect on insurance, psychological state, and impact on family
- The risk of incidental findings

The complexity of a test result also necessitates the need for appropriate posttest genetic counseling. This is to enhance patient understanding, minimize misunderstanding, and to help the patient cope with the outcome. The points that are usually covered include:

- The results of the genetic test and the interpretation of these results;
- The implications of the test results to the patient and his/her family;
- Where the patient is found to have a condition or genetic variant/change, the treatment and management options of the condition or genetic variant/change, and their potential outcomes;
- Any psychological, social, and ethical issues or concerns;
- Where relevant, the consideration for testing of family members' carrier status and/or variant status for confirmation of the patient's condition;
- Where relevant, to cover other complicated matters such as the risk of transmission and family planning.

## Conclusion

The accelerated discovery of targeted therapies and treatment options for genetic neuromuscular disorders has made their diagnosis imperative for individualized precision medicine. Gene panels are suitable for the simultaneous analysis of multiple genes in the genetically heterogenous group of neuromuscular diseases, reducing diagnostic delay and cost. Most gene panels are NGS based. Techniques such as repeat analysis, Sanger fill-in, and CNV analysis further enhance the high depth of coverage of gene panels. The optimal usage of gene panels, however, depends on the ordering clinician's clinical expertise in determining the proband's phenotype, understanding the benefits and limitations of a gene panel, and selecting an appropriate gene panel as part of a personalized diagnostic process. Expertise in the interpretation and clinical correlation of genetic test results is also necessary. The most complete and accurate interpretation of the results of the gene panel is best achieved with the help of a multidisciplinary team. As cost declines for WES, WGS, and transcriptome studies, these may become more widely used. In the meantime, gene panels generally remain as the first-tier, most readily available tool in delivering individualized precision medicine to neuromuscular patients. The complex nature of genetic testing can make clinician feel out of their league. If so, collaboration and discussion with relevant experts who can help you with the genetic testing while you continue exercising your expertise in managing the patient is recommended.

## Author contributions

KN wrote the first draft of the manuscript. DG, H-LC, and AC wrote sections of the manuscript. All authors contributed to manuscript revision, read, and approved the submitted version.

## Conflict of interest

The authors declare that the research was conducted in the absence of any commercial or financial relationships that could be construed as a potential conflict of interest.

## Publisher's note

All claims expressed in this article are solely those of the authors and do not necessarily represent those of their affiliated organizations, or those of the publisher, the editors and the reviewers. Any product that may be evaluated in this article, or claim that may be made by its manufacturer, is not guaranteed or endorsed by the publisher.

## Abbreviations

NGS: Next-generation sequencing
WES: Whole exome sequencing
WGS: Whole genome sequencing
AANEM: American Association of Neuromuscular and Electrodiagnostic Medicine
SMA: Spinal muscular atrophy
LGMD: Limb girdle muscular dystrophy
ALS: Amyotrophic lateral sclerosis
MRI: Magnetic resonance imaging
CK: Creatine kinase
CNVs: Copy number variations
FSHD: Facioscapulohumeral muscular dystrophy
OPMD: Oculopharyngeal muscular dystrophy
MLPA: Multiplex ligation-dependent probe amplification
CGH: Cytogenetic genomic hybridization
DMD: Duchenne muscular dystrophy
mtDNA: Mitochondrial DNA
ACMG: American College of Medical Genetics and Genomics
VUS: Variant of unknown significance
AMP: Association for Molecular Pathology.
